# Supplementary material for: Hierarchical encoding of natural sound mixtures in ferret auditory cortex
Source: eLife. 2025 Sep 23;14:RP106628. doi: 10.7554/eLife.106628 (PMC12456947; doi:10.7554/eLife.106628)
Supplement: Figure 1—source data 1. — Each column corresponds to a different run. [file elife-106628-fig1-data1.pdf]

Foregrounds

- Ferret (multiple) pup calls 1
- Ferret (multiple) pup calls 2
- Ferret fight calls
- Dormouse vocalization
- Orchestra music
- Biting and chewing
- Spanish speech
- Italian speech
- German speech
- Cello
- Chimes in the wind
- Chopping food

- Ferret (single) pup call 1
- Chicken
- Baby babble
- Ferret dooking
- Reggae music
- Finger tapping
- English speech
- Piano
- Saxophone
- Walking with heels
- French speech
- Russian speech

- Birds singing
- Ferret (single) pup call 2
- Dog barking
- Ferret fear vocalization
- Door opening and closing
- Chick vocalization
- Squeaky toy
- Baby crying
- Walking on leaves
- R&B music
- Violin
- Hindi speech

Backgrounds

- Applause - big room
- Crunching cellophane
- Fire
- Frogs 1
- Frying eggs
- Helicopter 1
- Metal lathe
- Radio static
- Rattlesnake rattle
- Scratching beard
- Teletype
- Waterfall

- Blender
- Castanets
- Drumroll
- Heavy rain on surface
- Jungle rain
- Pneumatic drills
- Rain
- Rain in woods
- Sander
- Small river
- Stream
- Wind blowing

- Air conditioner 1
- Air conditioner 2
- Construction site
- Fire alarm bell
- Frogs 2
- Helicopter 2
- Jackhammer
- Printing press
- Rhythmic applause
- Tambourine shaking
- Windshield wipers
- Motor engine
