# Supplementary material for: Hierarchical encoding of natural sound mixtures in ferret auditory cortex
Source: eLife. 2025 Sep 23;14:RP106628. doi: 10.7554/eLife.106628 (PMC12456947; doi:10.7554/eLife.106628)
Supplement: Figure 2—source data 1. — Values of background and foreground invariance for each ROI (MEG, dPEG, and VP), for different conditions: actual and predicted data, or restricted to voxels tuned to low (< 8 Hz) or high (> 8 Hz) temporal modulations. For each metric, we provide the median across voxels of each ROI for each animal (B, L, and R), as well as the p-values obtained to test the difference across pairs of regions. Metrics are also provided for the average across all animals (all). Significant p-values (p<0.05\begin{document}$p < 0.05$\end{document}) are highlighted in bold font. [file elife-106628-fig2-data1.pdf]

		background invariance	foreground invariance	predicted back-ground invariance	predicted fore-ground invariance	background invariance for low rates	foreground invariance for low rates	background invariance for high rates	foreground invariance for high rates
B	MEG	0.58	0.33	0.69	0.33	0.72	0.25	0.54	0.43
	dPEG	0.63	0.33	0.73	0.35	0.69	0.39	0.63	0.26
	VP	0.67	0.089	0.83	0.18	0.64	0.1	0.47	0.11
	p-val MEG-dPEG	**0.003**	0.975	**0.01**	**0.003**	0.378	**0.001**	**0.027**	**0.001**
	p-val dPEG-VP	0.29	**0.001**	**0.001**	**0.001**	0.09	**0.001**	0.06	0.366
L	MEG	0.41	0.43	0.67	0.3	0.47	0.46	0.4	0.47
	dPEG	0.58	0.3	0.66	0.28	0.61	0.24	0.57	0.41
	VP	0.8	0.22	0.77	0.22	0.86	0.2	0.74	0.25
	p-val MEG-dPEG	**0.001**	**0.001**	0.38	**0.001**	**0.001**	**0.001**	**0.001**	**0.015**
	p-val dPEG-VP	**0.001**	**0.001**	**0.001**	**0.008**	**0.001**	**0.005**	**0.001**	**0.002**
R	MEG	0.49	0.18	0.64	0.29	0.62	0.15	0.48	0.27
	dPEG	0.55	0.26	0.66	0.28	0.67	0.25	0.57	0.35
	VP	0.55	0.1	0.83	0.22	0.64	-0.06	0.49	0.31
	p-val MEG-dPEG	**0.005**	**0.002**	0.125	0.139	**0.017**	**0.001**	0.092	0.131
	p-val dPEG-VP	0.816	**0.001**	**0.001**	**0.001**	0.108	**0.001**	0.234	0.515
all	MEG	0.5	0.31	0.67	0.31	0.59	0.28	0.47	0.39
	dPEG	0.59	0.29	0.68	0.3	0.65	0.31	0.59	0.34
	VP	0.67	0.14	0.81	0.21	0.71	0.09	0.57	0.22
	p-val MEG-dPEG	**0.001**	0.14	**0.022**	**0.014**	**0.001**	0.561	**0.001**	**0.035**
	p-val dPEG-VP	**0.001**	**0.001**	**0.001**	**0.001**	**0.002**	**0.001**	0.585	**0.024**
